# Clinical outcomes and patient-reported joint noise after total knee arthroplasty using saddle-shaped rotating-platform and bi-cruciate-stabilized implant-surgical systems: a propensity score-matched cohort study

**DOI:** 10.1186/s42836-026-00426-2

**Published:** 2026-08-11

**Authors:** Masafumi Itoh, Umito Kuwashima, Junya Itou, Ken Okazaki

**Affiliations:** Department of Orthopedic Surgery, Tokyo Women’s Medical University, Shinjuku-ku, Tokyo, 162-8666 Japan

**Keywords:** Total knee arthroplasty, Rotating platform, Bi-cruciate-stabilized, Patient-reported joint noise, Forgotten Joint Score, Knee Injury and Osteoarthritis Outcome Score, Propensity score matching, Cementless fixation, Cemented fixation

## Abstract

**Background:**

Different posterior cruciate ligament-sacrificing total knee arthroplasty (TKA) systems use distinct stabilization concepts, but their comparative clinical outcomes, including joint noise and knee flexion, remain unclear. We aimed to compare clinical outcomes, including noise and flexion, between the saddle-shaped rotating platform (ROCC) and bi-cruciate-stabilized (BCS) TKA. We hypothesized that the ROCC implant-surgical system would be associated with lower patient-reported noise, whereas the BCS implant-surgical system would be associated with greater flexion.

**Methods:**

This retrospective propensity score-matched cohort study analyzed 556 consecutive primary TKAs for primary osteoarthritis (April 2017–April 2023). Patients with prior ligament surgery, additional ipsilateral procedures, or neurological disorders were excluded. Propensity score matching (1:1) for age, sex, body mass index, preoperative flexion, Knee Injury and Osteoarthritis Outcome Score (KOOS), and the University of California, Los Angeles activity score yielded 176 ROCC-BCS pairs. The primary outcome was KOOS-Pain score. Exploratory secondary outcomes included change in KOOS-Pain score, attainment of the KOOS-Pain minimal clinically important difference (MCID) (responder analysis), Forgotten Joint Score-12 (FJS), noise score (0–4), and maximum flexion. Spearman’s ρ was used to assess correlations among flexion, noise, KOOS, and FJS. Group values are reported as medians unless otherwise indicated.

**Results:**

Median follow-up was 36 months. For the primary outcome, KOOS-Pain was higher with ROCC than with BCS (94 versus 89; matched-pair difference, 4.2 points; 95% confidence interval, 1.4 to 8.3; *p* = 0.005), but the difference was below the 18-point MCID. In exploratory responder analysis, KOOS-Pain MCID attainment was higher with ROCC (90% versus 82%). Among secondary outcomes, FJS was higher with ROCC (56 versus 48), noise scores were lower with ROCC (0 versus 1), and flexion was higher with BCS despite identical medians of 130°. Noise correlated negatively with KOOS and FJS (*ρ* = –0.24 to –0.34), whereas flexion showed negligible correlations.

**Conclusions:**

ROCC was associated with modestly higher KOOS-Pain scores and lower patient-reported noise, but broader PROM findings were mixed. The KOOS-Pain difference was below the MCID, and its clinical relevance remains uncertain. These midterm findings may inform counseling on implant-surgical system trade-offs but require confirmation in broader prospective cohorts.

**Supplementary Information:**

The online version contains supplementary material available at 10.1186/s42836-026-00426-2.

## Introduction

Despite comparable survivorship across contemporary total knee arthroplasty (TKA) systems, some patients continue to report functional limitations and patient-perceived problems, including limited flexion, joint awareness, and mechanical noise [1]. Identifying implant and surgical strategies that better address these concerns has become increasingly important [2], particularly as annual TKA volumes in the United States are projected to exceed 1.2 million by 2040 [3–5]. Posterior cruciate ligament (PCL)-sacrificing designs include cruciate-sacrificing (CS) and post-cam-stabilized designs: CS does not rely on post-cam engagement but may limit flexion, whereas post-cam designs are intended to improve flexion yet have been associated with mechanical noise and patellar clunk [1, 6–9]. Joint noise after TKA has been associated with patient-reported outcomes, although its direct clinical significance remains uncertain [6, 8, 10–12].

**Fig. 1:**
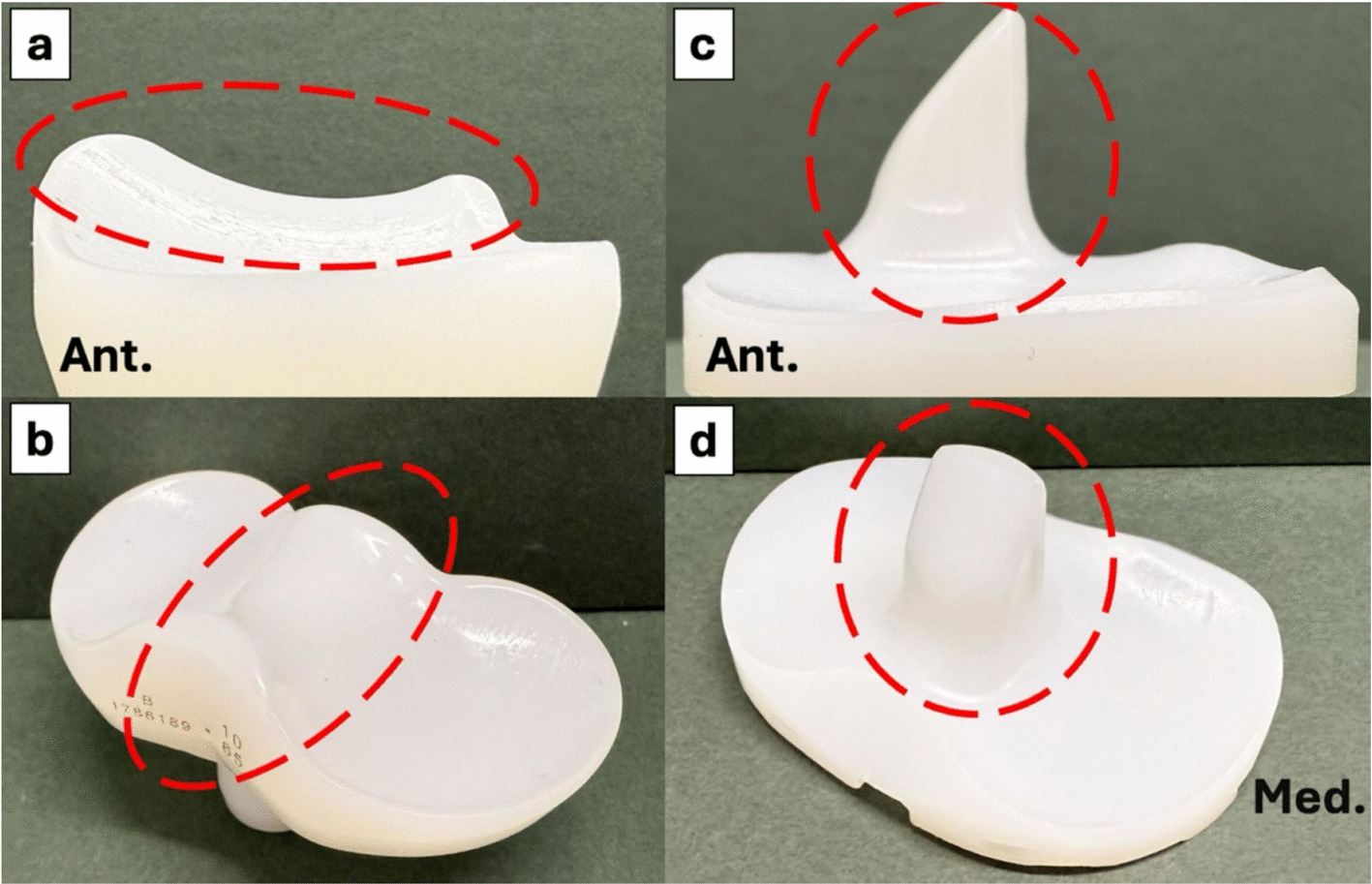
Insert design comparison highlighting key structural features of ROCC and BCS knee designs. (a) ROCC insert (lateral view); (b) ROCC insert (oblique superoanterior view) showing the saddle-shaped RP design with a built-in 7° posterior slope and rotational capability; (c) BCS insert (lateral view); (d) BCS insert (oblique superoanterior view) illustrating the antero-posterior post designed for cruciate-substituting function with biomimetic geometry and medial conformity. *Abbreviations*: ROCC, rotating concave-convex; BCS, bi-cruciate-stabilized. Red dashed circles highlight the key design features of each system (saddle-shaped prominence in ROCC, antero-posterior post in BCS). Ant., anterior; Med., medial

Recently, attention has shifted toward implant systems and surgical strategies intended to better replicate physiological knee kinematics while maintaining stability and function. The following two contrasting PCL-sacrificing designs exemplify distinct stabilization strategies. The rotating concave-convex (Vanguard ROCC; Zimmer Biomet), a CS-type TKA featuring a saddle-shaped rotating platform (RP), has demonstrated favorable long-term survivorship and promising short-term to mid-term outcomes [13–15]. The Journey II Bi-Cruciate-Stabilized (BCS; Smith & Nephew), an anterior–posterior cruciate-substituting TKA with dual post-cam mechanisms and biomimetic geometry, has been associated with excellent postoperative flexion and function [16]. However, head-to-head data comparing these two implant-surgical systems (Fig. 1) are lacking, limiting evidence-based selection between these systems.

Therefore, we aimed to compare outcomes between ROCC and BCS in matched cohorts, including postoperative flexion and noise. We hypothesized that the ROCC implant-surgical system would be associated with lower patient-reported noise and higher postoperative KOOS-Pain, whereas the BCS implant-surgical system would be associated with greater flexion.

## Materials and methods

### Study design, setting, and ethical considerations

This retrospective propensity score-matched cohort study was approved by the Ethics Committee of Tokyo Women’s Medical University (approval number: 4578) and conducted in accordance with the Declaration of Helsinki. Written informed consent was obtained from all patients.

**Fig. 2:**
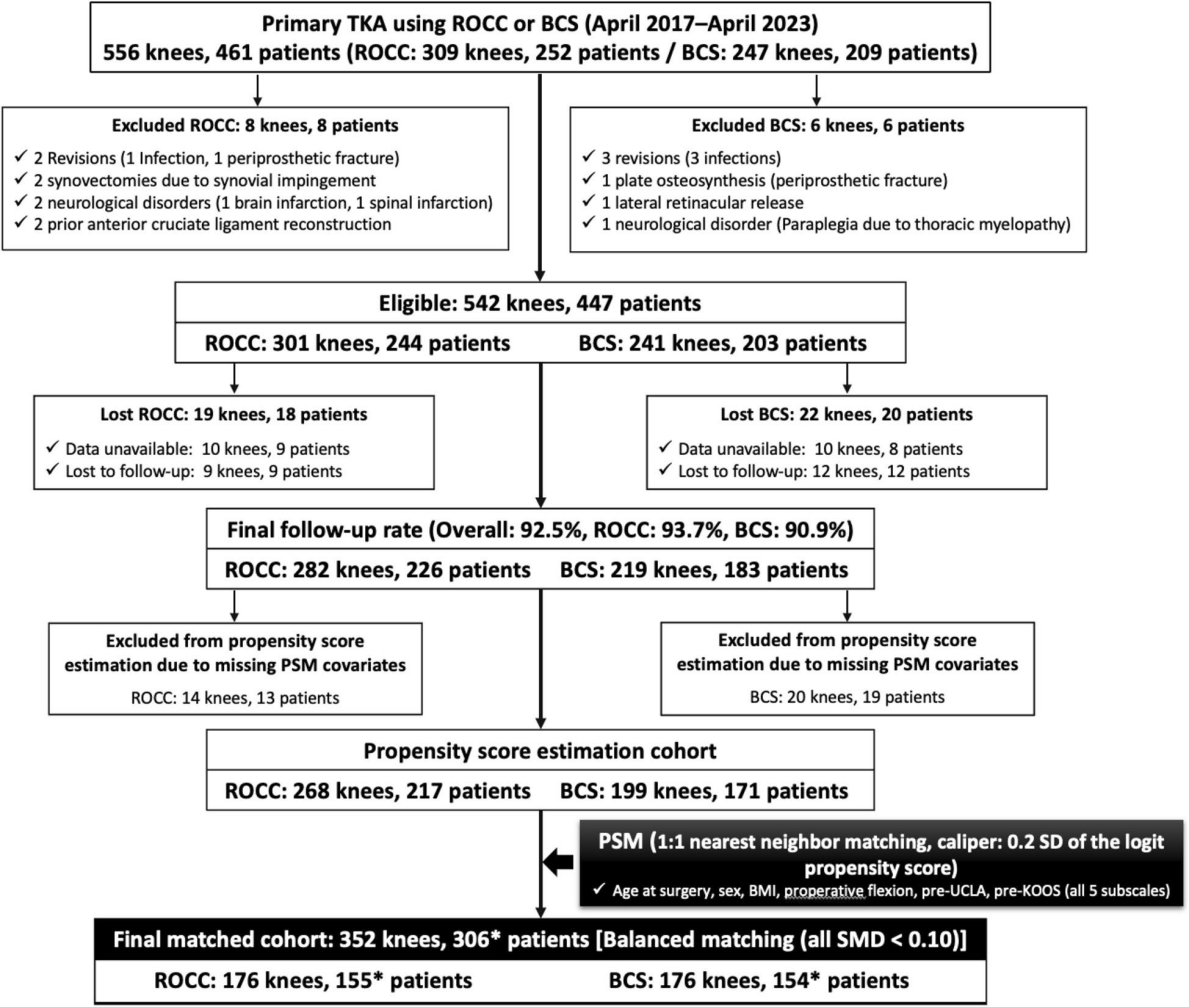
Study flow diagram for propensity score-matched ROCC and BCS-TKA cohorts. *Abbreviations*: TKA, total knee arthroplasty; ROCC, rotating concave-convex; BCS, bi-cruciate-stabilized; PSM, propensity score matching; SD, standard deviation; BMI, body mass index; UCLA, University of California, Los Angeles activity score; KOOS, Knee Injury and Osteoarthritis Outcome Score; SMD, standardized mean difference. After 1:1 nearest-neighbor matching within the prespecified caliper, 92 ROCC knees and 23 BCS knees were not retained. *Some patients contributed knees to both implant groups; therefore, group-specific patient counts do not necessarily sum to the overall patient count

Prospectively collected data on 556 knees from 461 consecutive patients who underwent primary TKA for osteoarthritis using either ROCC or BCS at our institution between April 2017 and April 2023 (Fig. 2) were reviewed.

Implant allocation was nonrandom and based on surgeon preference. Given surgeon preference-based allocation, we evaluated whether implant use varied by surgeon or year of surgery. Fixation method was determined by implant system (ROCC was cementless and BCS was cemented); we therefore considered both surgeon preference-based allocation and fixation method as design features that could confound between-group comparisons. All surgeries were performed by four knee specialists with equivalent experience in both implant techniques.

### Study population

The surgical indications and eligibility criteria are summarized in Table 1. As shown in Fig. 2, patients with major postoperative complications, neurological disorders, or insufficient follow-up were excluded per predefined criteria. Reoperations after the index TKA were recorded and summarized in the Supplementary Table S3. Reoperation rates were also calculated descriptively using the initial cohort before exclusions and propensity score matching (PSM) as the denominator.

**Table 1:** Eligibility criteria for primary TKA for osteoarthritis using ROCC and BCS designs

Selection criteria	Description
Indications for ROCC/BCS TKA	Coronal deformity: valgus ≤ 20°, varus ≤ 30˚ Functionally intact collateral ligaments No restriction on age, BMI, and activity level
Inclusion criteria	Minimum of 2-year follow-up Complete dataset available
Exclusion criteria	Previous ipsilateral ligament surgery Previous periarticular osteotomy on the ipsilateral knee Neurological disorders affecting knee function Additional surgery on the ipsilateral knee after TKA (e.g., deep infection and fracture)

*Abbreviations*: ROCC, rotating concave-convex; BCS, bi-cruciate-stabilized; TKA, total knee arthroplasty; BMI, body mass index

### Surgical procedure and postoperative management

Procedures were performed under general or spinal anesthesia with a pneumatic tourniquet. The surgical technique is outlined in Table 2. Antibiotics included cefazolin (2 g × 4) or clindamycin (600 mg × 3) for patients with penicillin allergies. All patients received standardized rehabilitation and a multimodal pain protocol administered by blinded physiotherapists.

**Table 2:** Surgical techniques and implant-surgical system characteristics of ROCC and BCS TKA

Surgical aspect	ROCC implant-surgical system	BCS implant-surgical system
Approach	Subvastus arthrotomy; tibia-first approach	Same
Alignment	Mechanical alignment	Same
Proximal tibial resection	Extramedullary guide; neutral coronal alignment; posterior slope set to 0°; insert has built-in 7° posterior slope	Extramedullary guide; neutral coronal alignment; posterior slope set to 3°; insert has 3° anterior slope medially and 1° posterior slope laterally
Distal femoral resection	Intramedullary guide; adjusted to individual femoral valgus angle	Same
Posterior femoral resection	Constant resection depth based on medial posterior condyle	Same
Femoral component rotation	Spacer block technique [14]	3–5° external rotation relative to the posterior condylar axis
Patellar management	Not resurfaced before December 2021; resurfaced thereafter	Resurfaced throughout the study period
Fixation method	Cementless	Cemented
Insert thickness	Selected to achieve full extension	Same
Drain placement	No drain	Same
Hemostatic agent	Intravenous tranexamic acid 1,000 mg at wound closure	Same

*Abbreviations*: TKA, total knee arthroplasty; ROCC, rotating concave-convex; BCS, bi-cruciate-stabilized

### Outcome measures and data collection

The primary outcome was the postoperative Knee Injury and Osteoarthritis Outcome Score Pain subscale (KOOS-Pain). Although the study hypotheses also addressed noise and flexion, pain relief represents the principal patient-centered goal following TKA. KOOS-Pain was selected a priori because it has a validated minimal clinically important difference (MCID), enabling robust sample size estimation [17, 18], whereas no established MCID was available for the noise scale used in this study.

All other endpoints were analyzed as exploratory secondary outcomes. Domain-specific MCIDs were 18 points for KOOS-Pain, 7 for Symptoms, 16 for Activities of Daily Living (ADL), and 17 for Quality of Life (QOL) [18]. No MCID has been established for the Sport/Rec subscale, which was reported descriptively. Joint awareness was assessed using the Forgotten Joint Score-12 (FJS), whose MCID is 13.7 points [19, 20]. For each KOOS subscale and for FJS, we assessed the following outcomes: (i) absolute postoperative score, (ii) change from baseline (Δ), and (iii) MCID attainment, defined as the proportion of patients whose Δ exceeded the relevant MCID (responder analysis) [21].

Patient-reported joint noise was evaluated separately for each operated knee, including bilateral cases, using a 5-point Likert scale based on the question: “When bending and extending your knee, do you notice a clicking or clacking noise that bothers you?” Response options were scored as 0 = never, 1 = almost never, 2 = seldom, 3 = sometimes, and 4 = mostly, with lower scores indicating less noise [8]. The item assessed perceived noise during knee flexion-extension regardless of weight-bearing status, and objective acoustic confirmation was not performed. Maximum passive knee flexion was measured in the supine position using a long-arm goniometer by a physiotherapist blinded to implant design.

Additional variables included age, sex, body mass index, follow-up duration, pre- and postoperative University of California, Los Angeles (UCLA) activity score [22], preoperative Kellgren–Lawrence grade, maximum extension, and hip–knee–ankle (HKA) angle. HKA was measured on standing long-leg radiographs by an independent surgeon and classified as valgus if > 180°. Preoperative assessments were performed within two weeks prior to surgery, whereas postoperative assessments, including noise, were performed at 1, 2, and 3 years postoperatively and biennially thereafter. The most recent complete dataset for each patient (median follow-up: 36 months) was used in the analysis.

### Sample size calculation

Sample size calculation for the primary outcome (KOOS-Pain) assumed an 11-point target difference, a standard deviation of 17.2 points, a two-sided α of 0.05, and 90% power [18, 23]. The 11-point target corresponded to approximately 61% of the reported KOOS-Pain MCID of 18 points and was selected as a statistically detectable difference based on the MCID-based planning framework [24], not as a redefinition of the MCID. This calculation required 52 knees per group; the final matched cohort included 176 pairs.

#### PSM

To balance patient characteristics between the ROCC and BCS groups, PSM was performed using variables known to influence TKA outcomes [25–27]: age at surgery, sex, body mass index, preoperative maximum flexion, preoperative UCLA score, and all 5 KOOS subscales. The preoperative UCLA score was entered as a numeric ordinal variable. Missing data for variables used in the propensity score model were handled by complete-case analysis. Knees with missing values in any of these variables were excluded from propensity score estimation and matching. Preoperative FJS was not included in PSM because it conceptually overlaps with preoperative KOOS subscales; inclusion of both was considered redundant and could reduce matching efficiency. Fixation method was determined by implant system; therefore, it was not modeled as an independent covariate in the propensity score. A 1:1 nearest-neighbor matching method without replacement was performed using a caliper width of 0.2 standard deviations of the logit of the propensity score [28]. Knees without an acceptable match within the prespecified caliper were not retained in the matched cohort, thereby restricting the matched cohort to the region of propensity score overlap. Balanced matching was defined as a standardized mean difference (SMD) < 0.10 for all matched variables [28].

### Data analysis

Normality was assessed using the Shapiro–Wilk test. Continuous variables were expressed as the median [interquartile range]. For matched ROCC and BCS pairs, paired tests were used to account for non-independence [28]: the paired t-test was used for normally distributed continuous variables, the Wilcoxon signed-rank test for non-normally distributed continuous variables and ordinal variables, including noise scores, and McNemar’s test for binary outcomes, including MCID attainment. For KOOS-Pain (primary outcome), we report the Hodges–Lehmann matched-pair difference (ROCC minus BCS) with a 95% confidence interval. To examine whether noise scores differed according to patellar resurfacing status, the Mann–Whitney U-test was used to compare noise scores between resurfaced and non-resurfaced ROCC cases. The correlations between postoperative outcomes (noise, flexion, KOOS subscales, and FJS) were assessed using Spearman’s ρ and categorized as negligible (0.00–0.19), weak (0.20–0.39), moderate (0.40–0.59), strong (0.60–0.79), or very strong (0.80–1.00) [29]. Noise-data availability was summarized by implant group and matched pair, and noise-data missingness was explored using logistic regression with implant group, calendar year, and follow-up duration as covariates. After PSM, no imputation was performed for outcome data. Outcomes were analyzed using available data, and the resulting analytic n is shown in the relevant tables. A two-sided *p*-value < 0.05 was used as the decision threshold for the prespecified primary outcome only. All other analyses were secondary and exploratory; *p*-values are reported as nominal and should be interpreted descriptively without multiplicity adjustment. As sensitivity analyses for KOOS-Pain, we fitted multivariable linear regression models to address potential surgeon-related and temporal confounding. These models included implant system and surgeon as covariates and additionally adjusted for either follow-up duration or calendar year. Matched-pair cluster-robust standard errors were used. To address bilateral knees, one knee per patient was randomly selected using a fixed seed and analyzed using the Wilcoxon rank-sum test with the Hodges–Lehmann estimate. Patient-level clustered bootstrap resampling with 10,000 samples was also applied to matched-pair KOOS-Pain differences calculated as ROCC minus BCS, and the mean difference was reported with a percentile-based 95% confidence interval. For exploratory adjusted analyses of secondary outcomes, postoperative FJS was modeled using linear regression adjusted for surgeon, follow-up duration, and calendar period, which was treated as a categorical variable. Noise score was analyzed both as a 0–4 score using the same linear regression framework and as an ordered 5-point outcome using ordinal regression. For each outcome, a second model additionally included patellar resurfacing. Matched-pair cluster-robust standard errors were used for the secondary-outcome linear models. Statistical analyses were performed using R version 4.0.3.

## Results

### Study cohort and baseline characteristics

PSM based on selected covariates yielded 176 matched pairs, with matching rates of 65.7% for ROCC and 88.4% for BCS; all matching variables were well balanced (SMDs < 0.10). The lower matching rate in the ROCC group reflected the larger number of eligible ROCC knees before matching. Median follow-up was 36.0 months in both groups (Table 3). Pre- and post-matching balance for baseline and clinically relevant preoperative variables is shown in the Supplementary Table S1 and Fig. S1. Implant use was similar across surgeons, whereas BCS was used more frequently in earlier years (Supplementary Table S2).

**Table 3:** Baseline characteristics and patient-reported outcome measures after PSM in patients undergoing ROCC or BCS-TKA

Factor	ROCC (*n* = 176)	BCS (*n* = 176)	SMD
^§^Female sex, % (n)	71.0 (125)	72.2 (127)	0.027
^§^Age at surgery, yrs	74.0 [68.0–78.0]	74.0 [69.0–78.3]	0.064
^§^BMI, kg/m^2^	25.5 [22.9–29.2]	25.8 [23.1–28.2]	0.031
Preoperative extension, °	5.0 [0.0–10.0]	5.0 [0.0–10.0]	0.043
^§^Preoperative flexion, °	125.0 [115.0–130.0]	125.0 [110.0–130.0]	0.054
^§^Preoperative UCLA	5.0 [3.8–6.0]	5.0 [3.0–6.0]	0.044
Preoperative KOOS			
^§^Pain	47.0 [32.5–58.0]	44.0 [35.3–58.0]	0.030
^§^Symptom	54.0 [39.0–71.0]	54.0 [43.0–67.9]	0.0003
^§^ADL	57.0 [42.5–69.0]	54.2 [44.0–68.0]	0.003
^§^Sport & Rec	15.0 [5.0–30.0]	15.0 [5.0–30.0]	0.032
^§^QOL	25.0 [12.5–37.5]	25.0 [12.5–31.3]	0.028
Preoperative FJS	14.6 [5.2–27.1]	12.5 [6.3–22.9]	0.112
Preoperative HKA, °	171.8 [168.0–176.0]	170.0 [165.0–175.0]	0.058
Kellgren–Lawrence grade	4.0 [4.0–4.0]	4.0 [4.0–4.0]	0.080
Follow-up, months	36.0 [24.6–38.9]	36.0 [24.6–40.8]	0.220

*Abbreviations*: PSM, propensity score matching; ROCC, rotating concave-convex; BCS, bi-cruciate-stabilized; SMD, standardized mean difference; BMI, body mass index; UCLA, University of California, Los Angeles activity score; KOOS, Knee Injury and Osteoarthritis Outcome Score; ADL, activities of daily living; QOL, quality of life; FJS, Forgotten Joint Score-12; HKA, hip–knee–ankle angle; SD, standard deviation

^§^Variables included in the PSM. Continuous variables are expressed as the median [interquartile range]. SMD < 0.10 indicates acceptable balance

In a descriptive comparison of the initial cohort before exclusions and PSM, 4 of 309 ROCC knees (1.3%) and 5 of 247 BCS knees (2.0%) underwent reoperation. Revision TKA was performed in 2 ROCC knees (0.6%; one infection and one periprosthetic fracture) and 3 BCS knees (1.2%; all for infection). Non-revision procedures comprised 2 ROCC synovectomies for synovial capsule impingement (0.6%) and, in BCS, one lateral retinacular release and one plate osteosynthesis for a periprosthetic fracture (0.8%). The synovectomies and lateral release were considered potentially implant-surgical system-related mechanical reoperations (ROCC, 0.6%; BCS, 0.4%). Details are provided in the Supplementary Table S3.

### Primary and secondary outcomes—KOOS

The primary outcome, postoperative KOOS-Pain, was higher with ROCC than with BCS (matched-pair difference, 4.2 points; 95% confidence interval, 1.4 to 8.3; *p* = 0.005), a difference smaller than the MCID. Improvement from baseline was also greater with ROCC (Δ 44.3 vs 39.2), and a higher proportion of ROCC patients achieved the KOOS-Pain MCID (90.3% vs 82.4%). Among other KOOS subscales, ADL and QOL scores and their changes from baseline were higher with ROCC. Scores for Symptoms and Sport/Rec and MCID attainment rates for non-Pain subscales were similar between groups (Table 4). These secondary KOOS findings were exploratory.

**Table 4:** KOOS-Pain (primary outcome) and other KOOS-based exploratory secondary outcomes in ROCC and BCS-TKA

Factor	ROCC (*n* = 176)	BCS (*n* = 176)	*p*-value
**Primary outcome**			
KOOS-Pain	94.4 [83.3–100.0]	88.9 [77.8–94.4]	0.005^*^
**Secondary outcomes**			
Other KOOS subscales			
Symptom	89.3 [82.1–96.4]	85.7 [78.6–96.4]	0.081^*^
ADL	89.0 [77.9–95.6]	83.8 [75.0–92.7]	0.015^*^
Sport & Rec	55.0 [30.0–80.0]	52.5 [30.0–75.0]	0.307^*^
QOL	75.0 [59.4–87.5]	68.8 [50.0–81.3]	0.009^*^
**ΔKOOS**			
Pain	44.3 [30.3–55.8]	39.2 [24.9–51.4]	0.017^†^
Symptom	29.0 [14.5–50.4]	28.9 [14.7–44.5]	0.407^*^
ADL	25.1 [17.2–43.1]	25.2 [16.1–35.5]	0.047^*^
Sport & Rec	35.0 [10.0–55.0]	30.0 [10.0–50.0]	0.170^†^
QOL	50.0 [31.3–62.5]	43.6 [25.0–56.3]	0.036^*^
**MCID attainment of KOOS, % (n)**			
Pain	90.3 (159)	82.4 (145)	0.043^‡^
Symptom	89.8 (158)	86.4 (152)	0.429^‡^
ADL	76.1 (134)	75.0 (132)	0.894^‡^
QOL	83.5 (147)	85.2 (150)	0.532^‡^

*Abbreviations*: ROCC, rotating concave-convex; BCS, bi-cruciate-stabilized; KOOS, Knee Injury and Osteoarthritis Outcome Score; ADL, activities of daily living; QOL, quality of life; MCID, minimal clinically important difference (Pain: 18, Symptoms: 7, ADL: 16, QOL: 17); SD, standard deviation; IQR, interquartile range

Continuous variables are expressed as the median [IQR]. Paired t test was used for variables meeting normality assumptions; otherwise, Wilcoxon signed-rank test was used. Δ indicates change from baseline; MCID attainment is the proportion of patients whose Δ exceeded the relevant MCID

^*^Wilcoxon signed-rank test; ^†^paired t test; ^‡^McNemar test

For KOOS-Pain (primary outcome), the matched-pair between-group difference (ROCC minus BCS) was estimated using the Hodges–Lehmann method: 4.2 points (95% confidence interval, 1.4 to 8.3; *p* = 0.005)

*p*-values for secondary outcomes are nominal and reported descriptively without multiplicity adjustment

KOOS-Pain findings were consistent in sensitivity analyses adjusting for surgeon and either follow-up duration or calendar year (Supplementary Table S4). Findings were also consistent in sensitivity analyses accounting for bilateral knees, including one-knee-per-patient analysis and patient-level clustered bootstrap analysis of matched-pair differences (Supplementary Table S5).

### Exploratory secondary outcomes

**Fig. 3:**
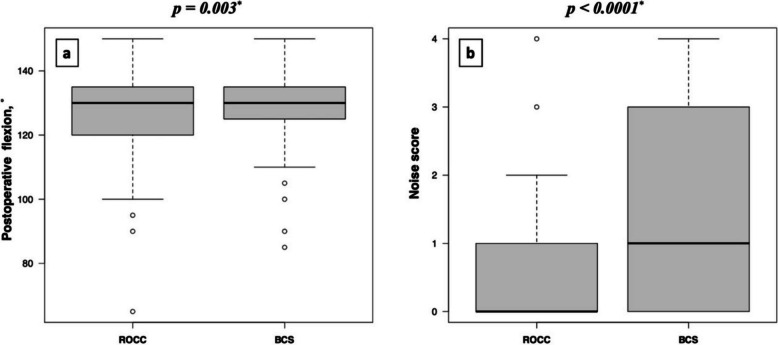
Comparison of postoperative flexion and noise scores between ROCC and BCS at final follow-up. **a** Postoperative maximum knee flexion (degrees). Median flexion was identical between groups (130°); **b** Patient-reported noise scores during knee flexion and extension (0 = never, 1 = almost never, 2 = seldom, 3 = sometimes, 4 = mostly); lower scores indicate less frequent joint noise. Box plots show the median (line), interquartile range (box), whiskers (1.5 × IQR), and outliers (circles). *Abbreviations:* ROCC, rotating concave-convex; BCS, bi-cruciate-stabilized; IQR, interquartile range. *Wilcoxon signed-rank test

FJS was higher with ROCC, whereas ΔFJS and MCID attainment were comparable between groups. Noise scores were lower with ROCC, but noise data were available for 144 ROCC knees and 130 BCS knees, with complete matched-pair noise data available for 107 pairs. Within ROCC, noise scores were similar between resurfaced and non-resurfaced patellae (Table 5). The noise-score distribution and matched-pair availability are shown in Supplementary Table S6A and S6B, respectively. Noise-data missingness was associated with implant group, calendar year, and follow-up duration (Supplementary Table S6C). In exploratory adjusted analyses using calendar period, additional adjustment for patellar resurfacing attenuated the postoperative FJS difference, whereas the higher noise score in BCS persisted but was also attenuated. Exploratory ordinal regression showed a consistent direction for higher noise-score categories in BCS (Supplementary Table S7). Postoperative knee flexion was higher with BCS, although both groups achieved the same median value of 130°. Other outcomes were comparable between groups (Table 5; Fig. 3).

**Table 5:** Exploratory secondary outcomes (FJS, flexion, and noise) and noise subgroup analysis in ROCC and BCS-TKA

Factor	ROCC (*n* = 176)	BCS (*n* = 176)	*p*-value
**Secondary outcomes**			
Postoperative FJS	56.3 [33.3–77.1]	47.9 [31.3–69.8]	0.039*
ΔFJS	39.6 [14.6–61.5]	31.3 [14.6–52.1]	0.166*
MCID attainment of FJS, % (n)	77.3 (136)	77.3 (136)	1.00^‡^
Postoperative flexion, °	130.0 [120.0–135.0]	130.0 [125.0–135.0]	0.003*
Noise score	0 [0–1] (*n* = 144)	1 [0–3] (*n* = 130)	< 0.0001*
**Other postoperative items**			
Age at final follow-up, yrs	76.4 [70.6–80.5]	77.2 [71.8–81.1]	0.200*
Postoperative extension, °	0.0 [0.0–0.0]	0.0 [0.0–0.0]	0.434*
Postoperative UCLA	6.0 [4.0–6.0]	6.0 [4.0–6.0]	0.695*
Postoperative HKA, °	178.6 [177.0–180.0]	179.0 [177.0–180.0]	1.000*
Subgroup analysis within the ROCC group only**			
Patellar management in ROCC	Patellar non-resurfacing (*n* = 113)	Patellar resurfacing (*n* = 63)	
Noise score	0 [0–1]	0 [0–1]	0.150^¶^

*Abbreviations*: ROCC, rotating concave-convex; BCS, bi-cruciate-stabilized; FJS, Forgotten Joint Score-12; UCLA, University of California, Los Angeles activity score; HKA, hip–knee–ankle angle; IQR, interquartile range; MCID, minimal clinically important difference

^*^Wilcoxon signed-rank test; ^‡^McNemar’s test; ^¶^Mann–Whitney *U* test. Data are expressed as the median [IQR]. Δ, change from baseline. Lower noise scores indicate less frequent noise. Noise scores were analyzed among knees with available noise data; noise-data availability and missingness are shown in Table S6. **Subgroup analysis limited to ROCC only. *p*-values are nominal and reported descriptively without multiplicity adjustment

### Correlations between outcomes

**Fig. 4:**
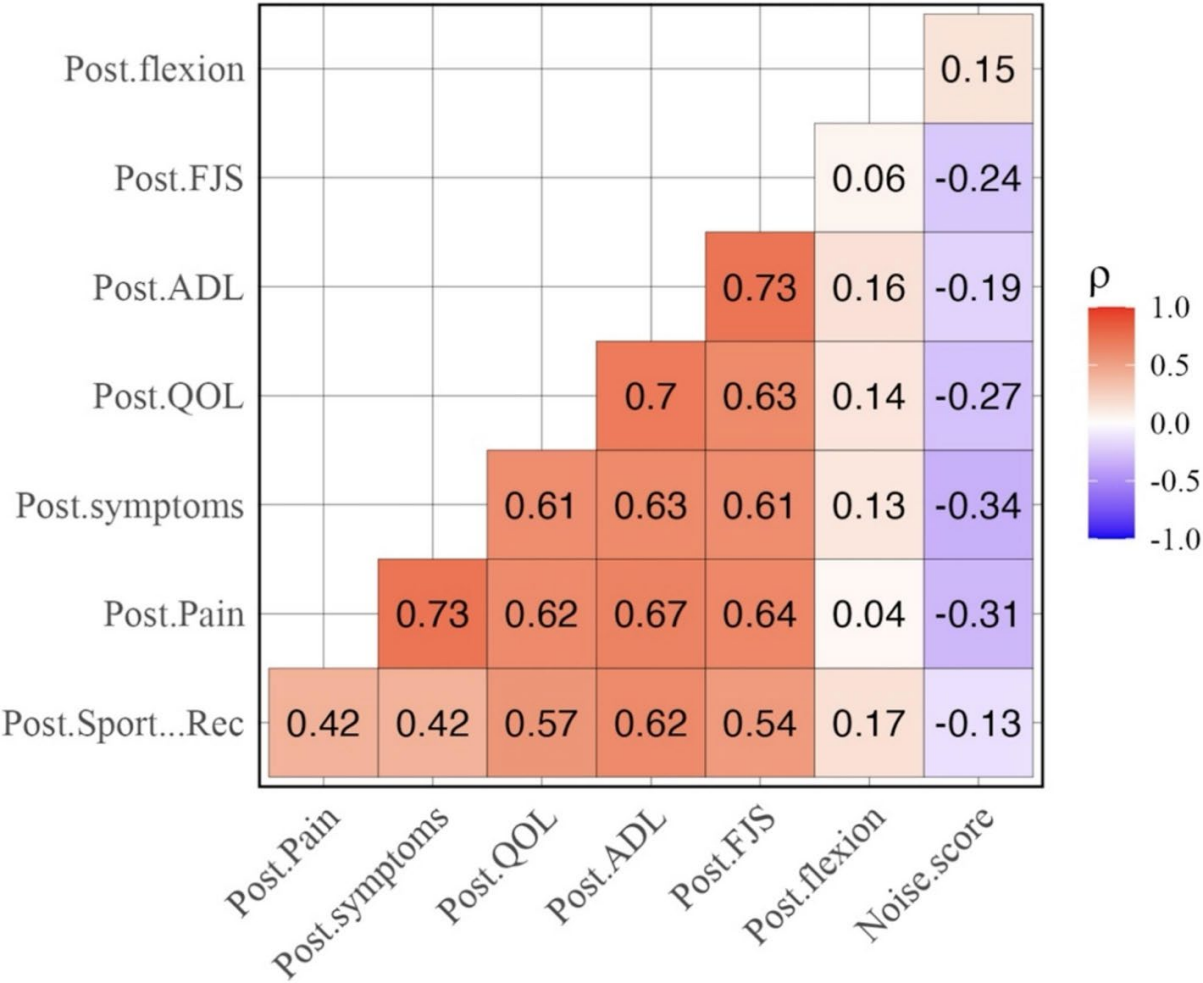
Heatmap of Spearman’s correlation coefficients (ρ) among noise, flexion, KOOS subscales, and FJS. *Abbreviations*: FJS, Forgotten Joint Score-12; ADL, activities of daily living; QOL, quality of life; KOOS, Knee Injury and Osteoarthritis Outcome Score. Correlation strength interpretation: *ρ* = 0.00–0.19, negligible; 0.20–0.39, weak; 0.40–0.59, moderate; 0.60–0.79, strong; 0.80–1.00, very strong

Noise showed weak negative correlations with KOOS-Pain, -Symptoms, -QOL, and FJS (*ρ* = − 0.24 to − 0.34), whereas knee flexion showed negligible correlations with patient-reported outcomes (*ρ* = 0.04–0.17). Correlations between KOOS subscales and FJS ranged from moderate to strong, with most being strong (*ρ* = 0.60–0.79) (Fig. 4).

## Discussion

The key findings of this study were: (1) the primary outcome, KOOS-Pain, was higher with ROCC (94.4 vs 88.9), with higher MCID attainment in exploratory responder analysis (90.3% vs 82.4%); (2) other patient-reported outcomes were mixed, with some exploratory secondary scores higher with ROCC but without consistent differences in change scores or MCID attainment; and (3) flexion was slightly higher with BCS, whereas noise scores were lower with ROCC, with noise showing weak negative correlations with patient-reported outcomes and flexion showing negligible correlations. Overall, the findings were consistent with our hypotheses regarding lower noise with the ROCC implant-surgical system and greater flexion with the BCS implant-surgical system, while the KOOS-Pain between-group difference was modest and smaller than the MCID. Regarding exploratory secondary outcomes, postoperative flexion was slightly greater with BCS than with ROCC, consistent with prior reports showing favorable flexion with BCS [16, 30]. However, both groups shared the same median flexion (130°), suggesting limited clinical relevance despite statistical significance. Similarly, Langlois et al. reported excellent flexion with ROCC (mean 127.7°) and suggested that design-related features, such as posterior condylar offset and patellofemoral tracking, may have contributed to this finding [25]. Subgroup analysis within the ROCC group showed no significant difference in noise between resurfaced and non-resurfaced patellae; however, this exploratory analysis does not exclude possible contributions from patellofemoral tracking or other technical factors. Previous studies have reported more frequent noise in posterior-stabilized than cruciate-retaining TKA designs (odds ratio, 2.5; 95% confidence interval, 1.8 to 3.7; *p* < 0.001) [8, 10, 31]. However, the present study did not include objective acoustic, kinematic, or radiographic assessment; therefore, the source of patient-reported noise and the relative contributions of implant geometry, component positioning, patellofemoral tracking, and soft-tissue balance remain uncertain.

Our finding that noise scores showed weak negative correlations with KOOS subscales and FJS aligns with previous reports indicating that persistent joint noise may increase patient anxiety and mistrust, and that both FJS and KOOS-JR scores improve as noise frequency decreases [6, 11]. By contrast, postoperative knee flexion showed no correlation with KOOS or FJS, possibly because the between-group difference in flexion was too small to be clinically meaningful. Prior studies suggest that patient-reported noise generally diminishes by 3–4 years postoperatively [6]. However, our study was not designed to evaluate within-knee longitudinal changes in patient-reported noise. As the clinical impact of noise may continue to evolve and its significance remains controversial [12], longer observation is warranted.

The BCS design substitutes both cruciate ligaments through a dual post-cam mechanism, yielding more physiologic kinematics than conventional posterior-stabilized TKAs [30]. However, its mechanical complexity has been associated with specific complications. Second-generation implants demonstrate a lower reoperation rate than the first (1.54 versus 2.28 per 100 component-years), although the earlier design was associated with iliotibial-band friction and post-cam dislocation due to an asymmetric post-cam and excessive lateral rollback; the shorter mean follow-up for the second generation (1.86 versus 6.23 years) warrants ongoing surveillance [32]. Despite design improvements and a more forgiving surgical technique, the guided motion system of second-generation BCS requires precise component alignment. Rotational mismatches as small as 2.8° can adversely affect outcomes [33]. Additionally, traction-related pain of the iliotibial band or lateral retinaculum during screw-home motion has been reported in approximately 2% of patients despite correct implant positioning [34]. By contrast, the RP design of ROCC inherently accommodates greater rotational variability [14], potentially offering greater tolerance for component rotation. However, RP designs require meticulous soft-tissue balancing given the risk of insert spin-out [35]. We experienced two ROCC cases requiring subsequent arthroscopic synovectomy for synovial capsule impingement, possibly due to impingement between the synovial capsule and the high-conformity insert. Most reoperations occurred before or around the first scheduled postoperative PROM assessment, and knees requiring reoperation were excluded from the matched PROM analyses (Fig. 2; Supplementary Table S3). Arthroscopic synovectomy for synovial capsule impingement in the ROCC group and lateral retinacular release for retinacular friction in the BCS group may represent implant-surgical system-related complications. Excluding reoperation cases may have overestimated implant performance and should be considered when interpreting outcomes in both groups.

Because implant type was linked to fixation method in this cohort, the observed differences should be interpreted as comparisons between implant-surgical systems rather than isolated implant-design effects. Therefore, cautious interpretation is warranted to avoid overstating the superiority of ROCC over BCS. The matched-pair KOOS-Pain difference (4.2 points) was statistically significant but smaller than the MCID, indicating a modest between-group difference with uncertain clinical relevance. Although KOOS-Pain MCID attainment was higher in the ROCC group, this responder analysis should be interpreted cautiously. Preoperative KOOS-Pain was included in the propensity score model and was well balanced after matching; nevertheless, responder proportions can still be influenced by baseline score distribution, ceiling effects, and regression to the mean. Therefore, the MCID responder analysis should be regarded as supportive and exploratory rather than definitive evidence of clinically meaningful superiority. Clear clinically meaningful superiority was not demonstrated across broader PROMs, including FJS change scores and FJS MCID attainment, likely reflecting ceiling effects or the multifactorial nature of TKA recovery [36]. Pain reduction should be interpreted within broader patient goals and implant selection. These findings suggest that differences between implant-surgical systems may be associated with patient-reported pain and noise.

PSM on key covariates, including demographics, activity level, preoperative KOOS subscales, and knee flexion, improved baseline balance between groups. The final matched cohort exceeded the calculated sample-size requirement for the prespecified primary statistical comparison of KOOS-Pain, a key patient-centered outcome following TKA.

This study has several limitations. Implant allocation was nonrandom and surgeon preference-based, and residual confounding related to unmeasured treatment factors, including soft-tissue balancing philosophy, alignment philosophy, and postoperative rehabilitation regimens, cannot be fully excluded despite PSM. Because fixation method was inseparable from implant type (cementless ROCC vs cemented BCS), this represents structural confounding, and its potential influence on patient-reported pain or noise cannot be disentangled. Although prior PSM work reported no meaningful differences in patient-reported outcomes by fixation [21], fixation remains inseparable from implant type in our study. Patellar management was also linked to implant system and calendar time: ROCC patellae were not resurfaced before December 2021, whereas both systems were resurfaced thereafter. A protocol-uniform sensitivity analysis was considered, but only 15 matched ROCC-BCS pairs had both procedures after December 2021, making reliable inference unstable. Therefore, the influence of patellar resurfacing on pain, anterior knee symptoms, noise, and joint awareness cannot be fully excluded. Consistent with this limitation, adjustment for patellar resurfacing attenuated the postoperative FJS difference, whereas the noise-score difference persisted but was also attenuated (Supplementary Table S7). In addition, BCS was used more frequently in earlier years; therefore, temporal bias, including learning-curve effects, cannot be fully excluded. However, implant use was similar across surgeons, and the KOOS-Pain association remained consistent in sensitivity analyses adjusting for surgeon and either follow-up duration or calendar year (Supplementary Table S4). Because some patients contributed both knees, within-patient correlation may have remained; however, KOOS-Pain findings were consistent in sensitivity analyses accounting for bilateral knees, including one-knee-per-patient analysis and patient-level clustered bootstrap analysis (Supplementary Table S5). Joint noise was assessed using a single patient-reported Likert item without standardized weight-bearing conditions or objective acoustic confirmation, limiting source attribution. Patient-reported noise may also have been influenced by subjective awareness, expectations, anxiety, or comparison with the contralateral knee. Noise data were unavailable for some knees, and noise-data missingness was associated with implant group, calendar year, and follow-up duration (Supplementary Table S6). Therefore, the noise findings should be interpreted as exploratory and potentially affected by non-random missingness. Detailed radiographic and technical parameters, including component rotation, achieved tibial slope, posterior condylar offset, joint-line change, patellar tilt or shift, component sizing, insert thickness, and intraoperative soft-tissue balance, were not uniformly available. Post hoc measurement in only a subset of patients could have introduced selection bias and measurement inconsistency. Because these unmeasured factors may influence flexion, patellofemoral mechanics, implant engagement, and patient-reported noise, the observed differences cannot be attributed solely to specific implant mechanisms. Because this retrospective study used the most recent complete postoperative dataset, outcomes were not assessed at a uniform fixed postoperative time point. Although the median follow-up was 36.0 months in both groups, follow-up duration remained imbalanced, and residual bias related to variable assessment timing cannot be fully excluded. The KOOS-Pain association remained consistent after adjustment for follow-up duration (Supplementary Table S4), but longitudinal modeling was not feasible because repeated postoperative PROMs were not uniformly available across outcomes. The 36-month follow-up provides only midterm insight; longer-term data are needed to evaluate implant durability and noise trends. The retrospective, single-center design from a specialist arthroplasty group and the predominantly older female cohort with relatively low BMI may limit generalizability. These findings may not translate directly to younger, heavier, more active, or more diverse TKA populations. Finally, as a single primary outcome was prespecified, multiplicity correction was not applied to secondary analyses, which should be interpreted descriptively. Future prospective studies incorporating standardized radiographic or CT-based technical assessment, gait analysis, objective noise assessment, and standardized expectation-setting protocols are warranted.

## Conclusions

In this selected propensity score-matched cohort, ROCC was associated with modestly higher KOOS-Pain scores and lower patient-reported noise than BCS, despite slightly lower postoperative flexion. However, the group-level KOOS-Pain difference was smaller than the MCID, and its clinical relevance remains uncertain. Findings across other KOOS subscales and joint awareness were mixed and did not demonstrate clear clinically meaningful superiority. Noise showed weak negative correlations with KOOS subscales and FJS. These midterm findings may help frame patient counseling regarding implant-surgical system trade-offs, but should be generalized cautiously given the single-center specialist setting and selected patient profile; confirmation in broader, more diverse prospective cohorts is needed.

## Supplementary Information

Supplementary Materials 1.

## Data Availability

The datasets generated and/or analyzed during the current study are not publicly available due to patient privacy considerations but are available from the corresponding author on reasonable request.
