# Supplementary material for: Clinical outcomes and patient-reported joint noise after total knee arthroplasty using saddle-shaped rotating-platform and bi-cruciate-stabilized implant-surgical systems: a propensity score-matched cohort study
Source: Arthroplasty. 2026 Aug 11;8:61. doi: 10.1186/s42836-026-00426-2 (PMC13459378; doi:10.1186/s42836-026-00426-2)
Supplement: Supplementary file 1 [file 42836_2026_426_MOESM1_ESM.docx]

**Supplementary Material**

**Clinical outcomes and patient-reported joint noise after total knee arthroplasty using saddle-shaped rotating-platform and bi-cruciate-stabilized implant-surgical systems: a propensity score-matched cohort study**

**Table S1.** Patient characteristics before and after propensity score matching

| **Factor** | **Before matching  (ROCC *n* = 268; BCS *n* = 199)** | | | **After matching (ROCC *n* = 176; BCS *n* = 176)** | | |
| --- | --- | --- | --- | --- | --- | --- |
|  | **ROCC** | **BCS** | **SMD** | **ROCC** | **BCS** | **SMD** |
| §Female sex, % (n) | 70.1 (188) | 69.8 (139) | 0.007 | 71.0 (125) | 72.2 (127) | 0.027 |
| §Age at surgery, yrs | 75.0 [68.0–79.0] | 74.0 [68.5–78.0] | 0.125 | 74.0 [68.0–78.0] | 74.0 [69.0–78.3] | 0.064 |
| §BMI, kg/m^2^ | 25.1 [22.7–28.9] | 25.8 [23.3–28.4] | 0.079 | 25.5 [22.9–29.2] | 25.8 [23.1–28.2] | 0.031 |
| Preoperative extension, degrees | 5.0 [0.0–10.0] | 5.0 [0.0–10.0] | 0.124 | 5.0 [0.0–10.0] | 5.0 [0.0–10.0] | 0.043 |
| §Preoperative flexion, degrees | 125.0 [120.0–135.0] | 125.0 [110.0–130.0] | 0.169 | 125.0 [115.0–130.0] | 125.0 [110.0–130.0] | 0.054 |
| §Preoperative UCLA | 4.0 [2.0–6.0] | 5.0 [4.0–6.0] | 0.267 | 5.0 [3.8–6.0] | 5.0 [3.0–6.0] | 0.044 |
| Preoperative KOOS |  |  |  |  |  |  |
| §Pain | 44.0 [28.0–56.0] | 47.0 [36.0–58.0] | 0.209 | 47.0 [32.5–58.0] | 44.0 [35.3–58.0] | 0.030 |
| §Symptom | 54.0 [38.2–71.0] | 54.0 [43.0–68.0] | 0.024 | 54.0 [39.0–71.0] | 54.0 [43.0–67.9] | 0.0003 |
| §ADL | 54.0 [38.0–69.0] | 56.0 [44.1–68.5] | 0.233 | 57.0 [42.5–69.0] | 54.2 [44.0–68.0] | 0.003 |
| §Sport & Rec | 15.0 [5.0–30.0] | 15.0 [5.0–30.0] | 0.017 | 15.0 [5.0–30.0] | 15.0 [5.0–30.0] | 0.032 |
| §QOL | 18.8 [12.5–31.2] | 25.0 [12.5–31.2] | 0.076 | 25.0 [12.5–37.5] | 25.0 [12.5–31.3] | 0.028 |
| Preoperative FJS | 12.5 [4.2–25.0] | 12.5 [6.2–22.9] | 0.024 | 14.6 [5.2–27.1] | 12.5 [6.3–22.9] | 0.112 |
| Preoperative HKA, degrees | 171.7 [167.2–175.9] | 169.0 [165.0–175.0] | 0.033 | 171.8 [168.0–176.0] | 170.0 [165.0–175.0] | 0.058 |
| Kellgren-Lawrence grade | 4.0 [4.0–4.0] | 4.0 [4.0–4.0] | 0.041 | 4.0 [4.0–4.0] | 4.0 [4.0–4.0] | 0.080 |

*Abbreviations*: PSM, propensity score matching; ROCC, rotating concave-convex; BCS, bi-cruciate-stabilized; SMD, standardized mean difference; BMI, body mass index; UCLA, University of California, Los Angeles activity score; KOOS, Knee Injury and Osteoarthritis Outcome Score; ADL, activities of daily living; QOL, quality of life; FJS, Forgotten Joint Score-12; HKA, hip–knee–ankle angle.

§Variables included in the propensity score matching. The prematching cohort represents knees with complete data for all variables entered into the propensity score model (ROCC, *n* = 268; BCS, *n* = 199), rather than all knees with final follow-up data. Continuous variables are expressed as the median [interquartile range], and categorical variables are expressed as % (n). SMD <0.10 indicates acceptable balance.

**Table S2.** Distribution of implant system by surgeon and year of surgery

**A. By surgeon**

| **Implant system** | **Surgeon A, % (n)** | **Surgeon B, % (n)** | **Surgeon C, % (n)** | **Surgeon D, % (n)** |
| --- | --- | --- | --- | --- |
| ROCC | 49.6 (58) | 46.9 (53) | 55.9 (33) | 50.8 (32) |
| BCS | 50.4 (59) | 53.1 (60) | 44.1 (26) | 49.2 (31) |
| Total | 100 (117) | 100 (113) | 100 (59) | 100 (63) |

**B. By year of surgery**

| **Implant system** | | **2017, % (n)** | **2018, % (n)** | **2019, % (n)** | | **2020, % (n)** | | **2021, % (n)** | | **2022, % (n)** | | **2023, % (n)** | |  |
| --- | --- | --- | --- | --- | --- | --- | --- | --- | --- | --- | --- | --- | --- | --- |
| ROCC | 4.2 (1) | | 33.9 (21) | | 56.0 (28) | | 58.5 (38) | | 78.3 (36) | | 55.7 (34) | | 40.9 (18) | |
| BCS | 95.8 (23) | | 66.1 (41) | | 44.0 (22) | | 41.5 (27) | | 21.7 (10) | | 44.3 (27) | | 59.1 (26) | |
| Total | 100 (24) | | 100 (62) | | 100 (50) | | 100 (65) | | 100 (46) | | 100 (61) | | 100 (44) | |

*Abbreviations*: ROCC, rotating concave-convex; BCS, bi-cruciate-stabilized

**Table S3.** Reoperations in the initial study cohort before exclusions and propensity score matching

|  | **ROCC (*N* = 309)** | **BCS (*N* = 247)** |
| --- | --- | --- |
| Knees with reoperation, n (%) | 4 (1.3) | 5 (2.0) |
| Potentially implant-surgical system-related mechanical reoperation, n (%) | 2 (0.6) | 1 (0.4) |
| Reoperation type (time from index TKA, mo) | Revision TKA (*n* = 2)   - Infection (*n* = 1): 5 mo - Periprosthetic fracture (*n* = 1): 2 mo   *Arthroscopic synovectomy for synovial capsule impingement (*n* = 2): 11 mo; 18 mo | Revision TKA for infection (*n* = 3): 2 mo; 3 mo; 7 mo  *Lateral retinacular release for retinacular friction (*n* = 1): 12 mo  Plate osteosynthesis for periprosthetic fracture (*n* = 1): 6 mo |

*Abbreviations*: TKA, total knee arthroplasty; ROCC, rotating concave-convex; BCS, bi-cruciate-stabilized.

N denotes knees in the initial cohort (≥2-year follow-up) before exclusions and propensity score matching (ROCC, 309; BCS, 247).

*Indicates potentially implant-surgical system-related mechanical reoperation.

**Table S4.** Sensitivity analyses of KOOS-Pain in the propensity score-matched cohort

**A. Adjusted for surgeon and follow-up duration (months)**

| **Covariate** | **β (95% CI)** | ***p*-value** |
| --- | --- | --- |
| Follow-up, months | 0.00 (−0.08 to 0.09) | 0.914 |
| Implant system (BCS vs ROCC)* | −4.5 (−7.6 to −1.3) | **0.005** |
| Surgeon B vs A | −3.0 (−6.5 to 0.5) | 0.097 |
| Surgeon C vs A | −2.6 (−6.2 to 0.9) | 0.147 |
| Surgeon D vs A | −1.5 (−5.6 to 2.6) | 0.467 |

**B. Adjusted for surgeon and calendar year**

| **Covariate** | **β (95% CI)** | ***p*-value** |
| --- | --- | --- |
| Calendar year | 0.2 (−0.6 to 1.0) | 0.549 |
| Implant system (BCS vs ROCC)* | −4.3 (−7.4 to −1.2) | **0.007** |
| Surgeon B vs A | −3.0 (−6.6 to 0.5) | 0.095 |
| Surgeon C vs A | −2.7 (−6.2 to 0.9) | 0.140 |
| Surgeon D vs A | −1.6 (−5.6 to 2.5) | 0.452 |

*Abbreviations:* KOOS, Knee Injury and Osteoarthritis Outcome Score; ROCC, rotating concave-convex; BCS, bi-cruciate-stabilized; CI, confidence interval.

*Negative values indicate lower (worse) KOOS-Pain in BCS than in ROCC.

In model B, calendar year was entered as a continuous variable. Multivariable linear regression with cluster-robust standard errors clustered by matched pair.

**Table S5.** Sensitivity analyses for postoperative KOOS-Pain accounting for bilateral knees

**A. One-knee-per-patient sensitivity analysis**

| ROCC, n | BCS, n | ROCC KOOS-Pain | BCS KOOS-Pain | Hodges-Lehmann difference (ROCC minus BCS) | *p*-value |
| --- | --- | --- | --- | --- | --- |
| 154 | 152 | 94.4 [83.3–100.0] | 88.9 [80.6–94.4] | 2.78 (0.00001 to 5.56) | 0.001 |

**B. Patient-level clustered bootstrap of matched-pair differences**

| Analysis | Estimate (ROCC minus BCS) | 95% bootstrap CI | Bootstrap samples |
| --- | --- | --- | --- |
| Mean matched-pair difference | 4.41 | 2.26 to 6.59 | 10,000 |

*Abbreviations*: KOOS, Knee Injury and Osteoarthritis Outcome Score; ROCC, rotating concave-convex; BCS, bi-cruciate-stabilized; CI, confidence interval.

In part A, values are shown as the median [interquartile range] unless otherwise indicated. One knee per patient was randomly selected using a fixed random seed. The Hodges–Lehmann difference is shown as ROCC minus BCS with the 95% CI; the Wilcoxon rank-sum test was used.

In part B, matched-pair differences were calculated as ROCC minus BCS. Patient-level clustered bootstrap resampling was performed using 10,000 samples, and the mean matched-pair difference is shown with the percentile-based 95% bootstrap CI.

**Table S6.** Patient-reported noise scores and noise-data availability at final follow-up

**A. Distribution of patient-reported noise scores**

| **Characteristic** | **ROCC (*n* = 176 knees)** | **BCS (*n* = 176 knees)** |
| --- | --- | --- |
| Knees with available noise data, n | 144 | 130 |
| Missing noise data | 32 (18.2%) | 46 (26.1%) |
| Noise score 0 (never) | 100 (69.4%) | 62 (47.7%) |
| Noise score 1 (almost never) | 24 (16.7%) | 16 (12.3%) |
| Noise score 2 (seldom) | 8 (5.6%) | 9 (6.9%) |
| Noise score 3 (sometimes) | 10 (6.9%) | 33 (25.4%) |
| Noise score 4 (mostly) | 2 (1.4%) | 10 (7.7%) |
| Any noise (score ≥1) | 44 (30.6%) | 68 (52.3%) |
| Noise at least sometimes (score ≥3) | 12 (8.3%) | 43 (33.1%) |

**B. Noise-data availability by matched pair**

| **Noise-data availability pattern** | **Matched pairs, n (%)** |
| --- | --- |
| Both ROCC and BCS available | 107 (60.8) |
| ROCC only available | 37 (21.0) |
| BCS only available | 23 (13.1) |
| Both missing | 9 (5.1) |

**C. Core logistic model for noise-data missingness**

| **Covariate** | **OR (95% CI)** | ***p*-value** |
| --- | --- | --- |
| Implant group (BCS vs ROCC) | 2.83 (1.47 to 5.57) | 0.002 |
| Calendar year | 2.41 (1.87 to 3.23) | <0.001 |
| Follow-up duration, months | 0.93 (0.88 to 0.98) | 0.006 |

*Abbreviations*: ROCC, rotating concave-convex; BCS, bi-cruciate-stabilized; OR, odds ratio; CI, confidence interval.

Values are shown as n (%) unless otherwise indicated.

In part A, percentages for noise score categories, any noise, and noise at least sometimes were calculated among knees with available noise data.

In part B, percentages were calculated among 176 matched pairs.

In part C, noise-data missingness was modeled as the dependent variable using logistic regression; OR >1 indicates higher odds of missing noise data. Noise score was evaluated using a patient-reported 5-point Likert scale: 0 = never, 1 = almost never, 2 = seldom, 3 = sometimes, and 4 = mostly. Any noise was defined as a noise score ≥1. Noise at least sometimes was defined as a noise score ≥3.

**Table S7.** Exploratory adjusted analyses of postoperative FJS and noise score

| **Outcome** | **Model** | **Effect estimate** | ***p*-value** |
| --- | --- | --- | --- |
| Linear regression models |  | β (95% CI) |  |
| Postoperative FJS | Surgeon + follow-up + calendar period | −8.2 (−14.5 to −1.9) | 0.011 |
| Postoperative FJS | Surgeon + follow-up + calendar period + patellar resurfacing | −3.7 (−12.7 to 5.3) | 0.419 |
| Noise score, 0–4 | Surgeon + follow-up + calendar period | 0.80 (0.50 to 1.10) | <0.001 |
| Noise score, 0–4 | Surgeon + follow-up + calendar period + patellar resurfacing | 0.57 (0.12 to 1.02) | 0.014 |
| Ordinal regression models |  | OR (95% CI) |  |
| Noise score, ordinal 0–4 | Surgeon + follow-up + calendar period | OR 3.16 (1.91 to 5.24) | <0.001 |
| Noise score, ordinal 0–4 | Surgeon + follow-up + calendar period + patellar resurfacing | OR 2.07 (1.01 to 4.24) | 0.047 |

*Abbreviations:* FJS, Forgotten Joint Score-12; ROCC, rotating concave-convex; BCS, bi-cruciate-stabilized; CI, confidence interval; OR, odds ratio.

Models adjusted for surgeon, follow-up duration (months), and calendar period. Calendar period was treated as a categorical variable. Patellar resurfacing was added in the second model for each outcome. β values indicate the adjusted difference for BCS versus ROCC. ORs from ordinal regression indicate the odds of being in a higher noise-score category for BCS versus ROCC. Cluster-robust standard errors were used for the linear models; ordinal regression results should be interpreted as exploratory because the proportional-odds assumption was not uniformly supported.

Noise score was evaluated using a patient-reported 5-point Likert scale: 0 = never, 1 = almost never, 2 = seldom, 3 = sometimes, and 4 = mostly.

**Fig. S1. Love plot of covariate balance before and after propensity score matching.** *Abbreviations*: BMI, body mass index; UCLA, University of California, Los Angeles activity score; KOOS, Knee Injury and Osteoarthritis Outcome Score; ADL, activities of daily living; Sport/Rec, sport and recreation; QOL, quality of life; FJS-12, Forgotten Joint Score-12; HKA, hip-knee-ankle angle; SMD, standardized mean difference. The dashed vertical line indicates an absolute SMD of 0.10, the prespecified threshold for acceptable balance. §Variables included in the propensity score model.
